# Spontaneous apoptosis of blood dendritic cells in patients with breast cancer

**DOI:** 10.1186/bcr1361

**Published:** 2005-12-16

**Authors:** Alberto Pinzon-Charry, Tammy Maxwell, Michael A McGuckin, Chris Schmidt, Colin Furnival, J Alejandro López

**Affiliations:** 1 Dendritic Cell and Cancer Laboratory, Queensland Institute of Medical Research, Brisbane, Queensland, Australia; 2 School of Medicine, University of Queensland, Brisbane, Queensland, Australia; 3 Mater Medical Research Institute, Brisbane, Queensland, Australia; 4 Wesley Medical Centre, Brisbane, Queensland, Australia

## Abstract

**Introduction:**

Dendritic cells (DCs) are key antigen-presenting cells that play an essential role in initiating and directing cellular and humoral immunity, including anti-tumor responses. Due to their critical role in cancer, induction of DC apoptosis may be one of the central mechanisms used by tumors to evade immune recognition.

**Methods:**

Spontaneous apoptosis of blood DCs (lineage negative HLA-DR positive cells) was assessed in peripheral blood mononuclear cells (PBMCs) using Annexin-V and TUNEL assays immediately after blood collection. The role of tumor products was assessed by culturing cells with supernatants derived from breast cancer cell lines (TDSN) or PBMCs (PBMC-SN, as a control). The capacity of DC stimulation to prevent apoptosis was assessed by incubating DC with inflammatory cytokines, poly I:C, IL-12 or CD40 ligand (CD40L) prior to culture with TDSN. Apoptosis was determined by flow cytometry and microscopy, and Bcl-2 expression determined by intracellular staining.

**Results:**

In this study we document the presence of a significantly higher proportion of apoptotic (Annexin-V^+ ^and TUNEL^+^) blood DCs in patients with early stage breast cancer (stage I to II; n = 13) compared to healthy volunteers (n = 15). We examined the role of tumor products in this phenomenon and show that supernatants derived from breast cancer lines induce apoptosis of blood DCs in PBMC cultures. Aiming to identify factors that protect blood DC from apoptosis, we compared a range of clinically available maturation stimuli, including inflammatory cytokines (tumor necrosis factor-α, IL-1β, IL-6 and prostaglandin (PG)E_2 _as a cytokine cocktail), synthetic double-stranded RNA (poly I:C) and soluble CD40 ligand. Although inflammatory cytokines and poly I:C induced robust phenotypic maturation, they failed to protect blood DCs from apoptosis. In contrast, CD40 stimulation induced strong antigen uptake, secretion of IL-12 and protected blood DCs from apoptosis through sustained expression of Bcl-2. Exogenous IL-12 provided similar Bcl-2 mediated protection, suggesting that CD40L effect is mediated, at least in part, through IL-12 secretion.

**Conclusion:**

Cumulatively, our results demonstrate spontaneous apoptosis of blood DCs in patients with breast cancer and confirm that *ex vivo *conditioning of blood DCs can protect them from tumor-induced apoptosis.

## Introduction

Dendritic cells (DCs) are bone marrow-derived leukocytes specialized in antigen presentation [1]. They play an essential role in initiating and directing cellular and humoral immunity, including antitumor responses. Tumor production of immunosuppressive factors (cytokines, arachidonic acid metabolites, glycosphingolipids, polyamines) with detrimental effects on DC maturation and function can significantly prevent the establishment of effective antitumor immune responses [2]. Recent evidence has indicated that induction of apoptosis in immune cells is yet another mechanism used by tumors to evade immune recognition [3]. Indeed, several studies have demonstrated that DCs undergo apoptosis after interacting with cancer cells or tumor-derived factors *in vitro *[4-7]. However, these studies have used DCs generated *in vitro *following prolonged culture with cytokines and cytokine-driven activity may not reflect the functional status of DC populations circulating *in vivo*.

*In vivo *circulating blood DCs are identified by their high expression of HLA-DR and lack of specific lineage markers (CD3, CD14, CD19, CD20, CD56 and CD34) found on other leukocytes [8]. DCs freshly isolated from blood offer the theoretical advantage of being in their natural state of differentiation, free from the influence of exogenous cytokines, more responsive and presumably capable of stimulating immune responses in a more physiological manner. Hence, there is active interest in using blood DCs as vectors for cancer immunotherapy, with preliminary reports confirming their clinical potential [9,10].

Several studies, however, have demonstrated severe phenotypic and functional impairment of DCs in patients with breast cancer [11,12]. Tumor-infiltrating DCs are neither mature nor activated [13,14] and blood DCs express low levels of co-stimulatory molecules [11,12] and IL-12 [15] and exhibit an impaired capacity to stimulate T-cells [11,12]. In this context, knowledge of the mechanisms responsible for tumor-induced DC defects in breast cancer is essential to overcome DC dysfunction and to harness their immunotherapeutic potential. Recent reports revealed spontaneous apoptosis of several subpopulations of peripheral blood mononuclear cells (PBMCs; T-cells, B-cells and monocytes) in patients with cancer [16-18]. Those findings together with the reported decreased DC function prompted us to assess the extent of spontaneous apoptosis in blood DCs from patients with breast cancer and to identify clinically available factors to protect blood DCs against tumor-induced apoptosis.

## Materials and methods

### Patients and donors

Thirteen female patients, 40 to 75 years of age, with histologically confirmed breast adenocarcinoma were enrolled in the study. All patients presented with early disease (stage I to II), were newly diagnosed and had received no prior cancer therapy. Staging was performed in accordance with the International Union Against Cancer, UICC TNM Classification [19]. In addition, 15 healthy female donors, 24 to 73 years of age, volunteered for the study and served as controls. The Australian Red Cross Blood Service, Brisbane, provided buffy coats. The research ethics committees of both the clinical (Wesley Medical Centre and Royal Brisbane and Women Hospital) and scientific (Queensland Institute of Medical Research) institutions approved the study protocols.

### Monoclonal antibodies, reagents and cytokines

The following monoclonal antibodies were used in this study: CD3, CD14, CD19, CD20, CD56, CD34, HLA-DR, CD80, CD86, tumor necrosis factor (TNF)-α and IgG1, IgG2a and IgG2b isotype controls from BD Pharmingen (BD Biosciences, San Jose, CA, USA); HLA-DR, CD40, CD83, CD19 and IgG1 isotype control from Beckman Coulter (Fullerton, CA, USA); and IL-10 and IL-12 from Caltag laboratories (Burlingame, CA, USA). All antibodies were used as the fluorescein isothiocyanate (FITC), phycoerythrin (PE), biotin, allophycocyanin (APC) or PE-Cy5 conjugate. The PE-conjugated Bcl-2 antibody reagent kit was purchased from BD Pharmingen. Complete media included RPMI 1640 supplemented with 10% fetal calf serum, penicillin (100 u/ml), streptomycin (100 μg/ml), L-glutamine (2 mM), HEPES (25 mM) and non-essential amino acids all purchased from Gibco Life Technologies (Gaithersburg, MD, USA). The combination of pro-inflammatory cytokines consisted of IL-1β (10 ng/ml), IL-6 (10 ng/ml) and TNF-α (10 ng/ml) obtained from R&D systems (Minneapolis, MN, USA) plus Prostaglandin E2 (PGE2, 1 μg/ml) from Sigma (St Louis, MI, USA). Double stranded RNA (poly I:C, 50 μg/ml) [20] was purchased from Sigma; IL-12 (100 ng/ml) was purchased from Mabtech (Stockholm, Sweden) and soluble human recombinant CD40-ligand (CD40L; 2 μg/ml) was kindly provided by Amgen (Seattle, WA, USA).

### Determination of apoptosis

Venous blood obtained from patients and volunteers was collected in heparinized tubes. Samples were processed and PBMCs recovered by Ficoll-Hypaque centrifugation. To determine the proportion of apoptotic cells, Annexin-V binding and TUNEL assays were performed after blood collection. In all experiments, each patient was tested in parallel with at least one healthy volunteer. Annexin-V binding assays were performed using the Annexin-V kit (BD Pharmingen). Briefly, PBMCs were adjusted to 10^6 ^cells/ml and stained with a mixture of lineage markers (CD3, CD14, CD19, CD20, CD56, CD34) and HLA-DR for 20 minutes at 4°C. CD34 was added to the lineage mixture to exclude circulating hematopoietic stem cells. Cells were washed and resuspended in binding buffer prior to incubating with Annexin-V and 7-Aminoactinomycin D (7-AAD) for 15 minutes at room temperature. Samples were analyzed by fluorescence activated cell sorting (FACS) within one hour of staining. The TUNEL assay was performed using the Apo-BrDU-Red DNA fragmentation assay kit (BioVision, Mountain View, CA, USA). As above, PBMCs were adjusted to 10^6 ^cells/ml, stained for surface markers, fixed with 1% (w/v) paraformaldehyde and resuspended in ice-cold 70% ethanol for 30 minutes prior to incubation with DNA labeling and antibody solutions according to the manufacturer's instructions. Samples were analyzed within one hour of staining. In all experiments, 5 to 10 × 10^5 ^events were collected within the mononuclear cell gate. Data were acquired on a FACS Calibur flow cytometer and analyzed using CellQuest 3.1 (BD Bioscience), FloJo (TreeStar, San Carlos, CA, USA) or Summit (DakoCytomation, Fort Collins, CO, USA) software.

### Tumor-derived supernatants

The breast cancer cell lines MCF7, MB435 and SKBR3 were sourced from the American Type Culture Collection (ATCC, Manassas, VA, USA). The MA11 line was a generous gift of Dr Phil Rye (Norwegian Radium Hospital, Oslo, Norway). Tumor-derived supernatants (TDSN) were prepared by seeding 10^7 ^tumor cells in 20 ml of complete medium and incubating for 72 h. Before passage, culture supernatants were collected, centrifuged to remove cells, dialyzed against fresh medium (24 to 48 h at 4°C in the dark) to replenish nutrients and stored at -20°C. Dialysis membranes (membra-cell, Polylabo, Strasborg, France) with a molecular weight cut-off of 10,000 to 14,000 were used. For each experiment, PBMCs were cultured (10^7 ^cells/ml) for 24 h in the presence of 50% (v/v) TDSN or PBMC-conditioned supernatant (PBMC-SN) as a control. Cells were harvested, washed and apoptosis in lineage negative HLA-DR positive (Lineage (Lin)^-^HLA-DR^+^) cells determined using Annexin-V binding and TUNEL assays. For morphological analysis following culture with TDSN, viable (7-AAD negative) Lin^-^HLA-DR^+ ^cells were sorted (99% purity) using a MoFlo Sorter (DakoCytomation), resuspended in complete medium and cyto-centrifuged. Histology was performed on cytospin preparations made by seeding 2 to 3 × 10^4 ^sorted cells onto a glass slide. These were air-dried and stained using May-Grunwald-Giemsa.

### Phenotypic maturation, cytokine secretion and Bcl-2 expression

Four-color flow cytometry was used to analyze the phenotype and cytokine secretion of Lin^-^HLA-DR^+ ^cells. Briefly, PBMCs were cultured (10^7 ^cells/ml) in 6-well plates for 18 to 36 h in complete medium in the presence of inflammatory cytokines (a cytokine cocktail (CC) containing IL-1β (10 ng/ml), IL-6 (10 ng/ml), TNF-α (10 ng/ml) plus PGE_2 _(1 μg/ml)), poly I:C (50 μg/ml) or CD40L (2 μg/ml) and subsequently stained for flow cytometric analysis. Doses and incubation times were optimized in preliminary experiments. For cytokine secretion, 10^7 ^PBMCs were cultured with the CC, poly I:C or CD40L (in addition to IFN-γ and IL-1β) in the presence of brefeldin-A (10 μg/ml; Sigma). Cells were stained for surface markers, fixed with 1% w/v paraformaldehyde and stained with cytokine-specific monoclonal antibodies (TNF-α, IL-10 and IL-12) in 0.2% w/v saponin/PBS at 4°C overnight. For determination of Bcl-2 expression, 10^6 ^PBMCs were stained for surface markers (CD3, CD14, CD19, CD20, CD56, CD34 and HLA-DR), fixed with 1% w/v paraformaldehyde and stained with anti-Bcl-2 or isotype control in 0.2% w/v saponin/PBS at 4°C overnight. In all experiments, 5 to 10 × 10^5 ^events were collected within the mononuclear cell gate. Data were acquired on a FACS Calibur flow cytometer and analyzed using CellQuest 3.1 (BD Bioscience), FloJo (TreeStar) or Summit (Cytomation) software.

### Statistical analysis

Comparisons of samples to establish statistical significance were determined by the two tailed Students' t-test or one way analysis of variance (ANOVA) followed by Bonferroni's comparison test. Results were considered to be statistically significant when the *p*-value was <0.05.

## Results

### Spontaneous apoptosis of blood dendritic cells in patients with breast cancer

**Figure 1:**
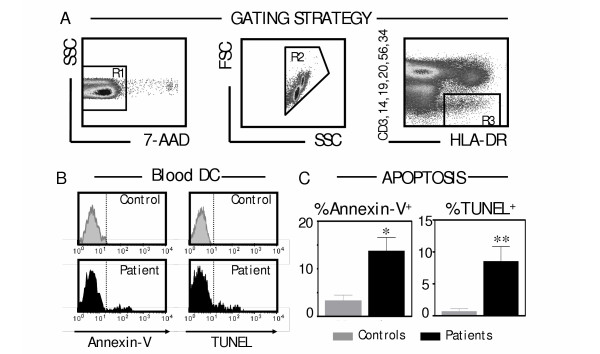
Spontaneous apoptosis of blood dendritic cells (DCs) in patients with breast cancer. Peripheral blood mononuclear cells isolated from patients with breast cancer (stage I to II; n = 13) and age-matched healthy volunteers (n = 15) were analyzed for apoptosis by flow cytometry. **(a)**In order to include all apoptotic cells and avoid debris, gating was set to include only 7-AAD negative events (R1) which were then visualized and re-gated on forward (FSC) and side scatter (SSC) characteristics (R2). Within this mononuclear cell population, blood DCs were identified as lineage negative HLA-DR positive cells (R3). Representative dot plots are shown. **(b)**Apoptosis in blood DCs from patients (black) and healthy volunteers (grey) was determined by two different methods, Annexin-V binding and TUNEL assays. In all experiments, each patient was tested in parallel with at least one healthy volunteer. Representative histograms are shown. **(c)**Summary of apoptosis data. The proportions of Annexin-V^+ ^and TUNEL^+ ^blood DCs estimated for all patients (black) and healthy volunteers (grey) are shown accordingly. Error bars indicate the standard error of the mean. Statistically significant differences between controls and patients are indicated as: *, *p *< 0.05; **, *p *< 0.01.

In accordance with the published literature [11-14], we also confirmed that there is significant DC dysfunction in patients with breast cancer prior to therapy (Additional file 1). Given that apoptotic DCs are ineffective at inducing immunity, here we examined the presence of apoptotic DCs obtained from patients with breast cancer (stage I to II; n = 13) compared to age-matched healthy females (n = 15). In order to include all cells undergoing apoptosis, gating was set as described in Fig. 1a. This strategy of double gating confirmed the viability of all cells while eliminating acellular debris. Blood DCs were identified as Lin^-^HLA-DR^+ ^cells and apoptosis was assessed using Annexin-V and TUNEL assays. The minimal proportion of spontaneously apoptotic blood DCs in healthy volunteers was significantly increased in patients with breast cancer (*p *< 0.05 and *p *< 0.01, respectively) (Fig. 1b,c).

### Apoptosis in blood dendritic cells is induced by tumor derived supernatants

**Figure 2:**
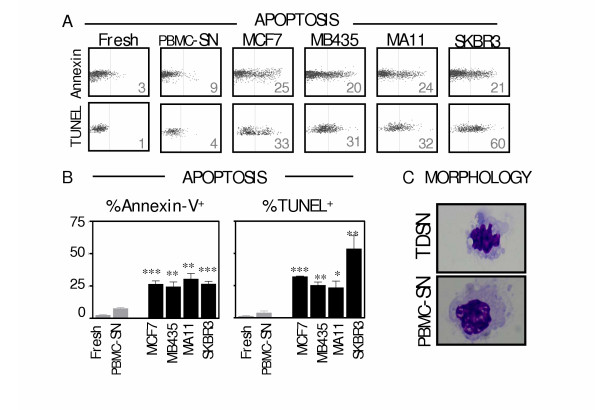
Apoptosis in blood dendritic cells (DCs) is induced by breast tumor-derived supernatants. **(a)**Apoptosis in blood DCs was determined by Annexin-V binding and TUNEL assays following culture (24 h) in the presence of 50% (v/v) peripheral blood mononuclear cell (PBMC)-derived supernatant (PBMC-SN) or tumor-derived supernatants (TDSN; MCF7, MB435, MA11 and SKBR3). Representative dot plots (SSC on y-axis versus apoptosis on x-axis) are shown with numbers indicating the percentage of apoptotic cells. **(b)**Summary of apoptosis data. The proportions of Annexin-V^+ ^and TUNEL^+ ^blood DCs represent the average ± standard error of the mean of five independent experiments for which statistically significant differences compared to PBMC-SN are shown: *, *p *< 0.05; **, *p *< 0.01; ***, *p *< 0.001. **(c)**For morphological analyses (original magnification times 1,000), 50 cells were assessed in three separate fields. In contrast to PBMC-SN, cultures incubated with TDSN (MA11) frequently demonstrated features of apoptotic death in blood DCs (reduction in cell volume and nuclear size, cytoplasmic blebbing, cell membrane convolutions and formation of apoptotic bodies). Three independent experiments with similar findings were performed.

Given that significantly less apoptosis was observed in samples from healthy volunteers, we hypothesized that tumor products were responsible for the elevated proportion of apoptotic blood DCs in patients with breast cancer. To more accurately examine this hypothesis, we used an *in vitro *model of blood DC culture in the absence of exogenous cytokines [21]. For this purpose, PBMCs were incubated in the presence of TDSN and apoptosis measured in Lin^-^HLA-DR^+ ^cells. Incubation with PBMC-SN served as a control. All supernatants were filtered and dialyzed against fresh medium prior to use. This approach excluded the possibility of apoptosis induced by nutrient depletion. Analysis of Annexin-V binding and TUNEL assays in different cultures revealed minimal apoptosis on fresh samples and less than 10% apoptosis following culture with PBMC-SN (Fig. 2a,b). On the other hand, incubation with TDSN resulted in a significant (*p *< 0.05) increase in the proportion of apoptotic Lin^-^HLA-DR^+ ^cells. Moreover, morphological evaluation frequently demonstrated features of apoptotic death in cultures containing TDSN (Fig. 2c). These data suggest that factors derived from breast cancer cell lines induce apoptotic death of Lin^-^HLA-DR^+ ^cells.

### CD40 stimulation protects blood dendritic cells against TDSN-induced apoptosis

**Figure 3:**
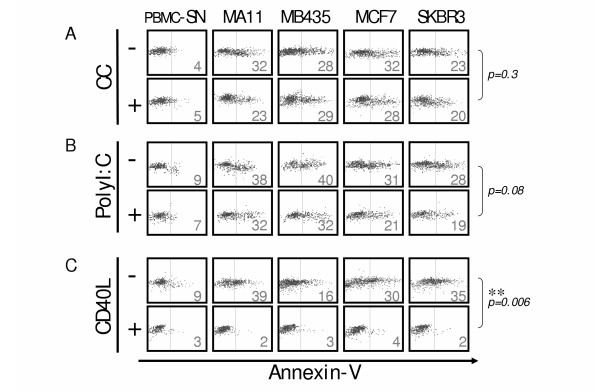
CD40 stimulation protects blood dendritic cells (DCs) against tumor-derived supernatant (TDSN)-induced apoptosis. TDSN-induced apoptosis in blood DCs was determined by Annexin-V binding following incubation (24 h) in the presence or absence of: **(a)**a combination of inflammatory cytokines (tumor necrosis factor-α, IL-1β, IL-6 and prostaglanding (PG)E_2 _as a cytokine cocktail (CC)); **(b)**synthetic double-stranded RNA (poly I:C); or **(c)**CD40 ligand (CD40L) prior to culture (24 h) with 50% (v/v) peripheral blood mononuclear cell-conditioned supernatant (PBMC-SN) or TDSN (MA11, MB435, MCF7 and SKBR3). Representative dot plots (SSC on y-axis versus apoptosis on x-axis) are shown with numbers indicating the percentage of apoptotic cells. Five independent experiments were performed for which *p*-values are indicated.

It has recently been demonstrated that protection of DCs from apoptosis can improve antitumor immunity *in vivo *[22]. Therefore, the identification of factors that protect blood DCs against tumor-induced apoptosis could enhance their potential for immunotherapy. Analysis of Annexin-V binding revealed that pre-incubation with a CC did not decrease apoptosis in blood DCs whereas poly I:C stimulation induced a modest (not significant) reduction in apoptosis (Fig. 3). In contrast, incubation with CD40L demonstrated a significant (*p *< 0.05) decrease of TDSN-induced apoptosis to levels seen in control cultures. These data show that CD40 stimulation leads to increased resistance of blood DCs to TDSN-induced apoptosis.

### Effects of IL-12 on TDSN-induced apoptosis

**Figure 4:**
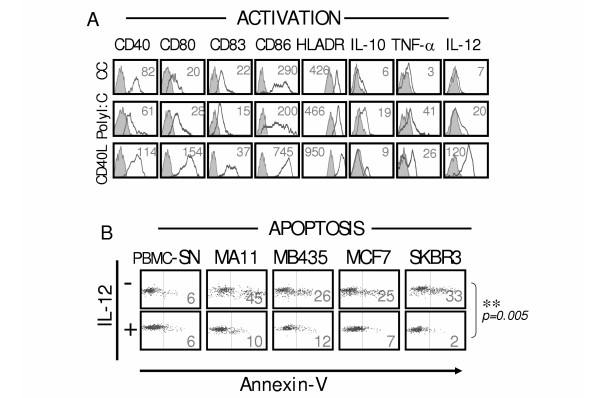
Protective effect of IL-12 on tumor-derived supernatant (TDSN)-induced apoptosis. **(a)**Expression of HLA-DR and co-stimulatory molecules (CD40, CD80, CD83 and CD86) as well as secretion of cytokines (tumor necrosis factor (TNF)-α, IL-10 and IL-12) was determined for blood DCs following stimulation (24 h) with a cytokine cocktail (CC), synthetic double-stranded RNA (poly I:C) or CD40 ligand (CD40L) as described in Materials and methods. Histograms indicate expression in the absence (shaded) or presence (non-shaded) of stimulation. Numbers indicate delta mean fluorescence intensity (ΔMFI, stimulated cells minus unstimulated cells) and are representative of five independent experiments. **(b)**TDSN-induced apoptosis in blood DCs was determined by Annexin-V binding following incubation (24 h) in the presence or absence of exogenous IL-12 prior to culture (24 h) with 50% (v/v) peripheral blood mononuclear cell-conditioned supernatant (PBMC-SN) or TDSN (MA11, MB435, MCF7 and SKBR3). Representative dot plots (SSC on y-axis versus apoptosis on x-axis) are shown with numbers indicating the percentage of apoptotic cells. Five independent experiments were performed for which the *p*-value is indicated.

Due to the differential effects of inflammatory mediators on DC apoptosis, we assessed the functional maturation induced on Lin^-^HLA-DR^+ ^cells following stimulation with a CC, poly I:C or CD40L. All maturation stimuli induced significant increases in the expression of HLA-DR and co-stimulatory molecules (CD40, CD80, CD83 and CD86) (Fig. 4a). The cytokine secretion profile, however, was markedly different between stimuli. While the CC induced minimal cytokine secretion, poly I:C induced moderate secretion of TNF-α as well as IL-12. On the other hand, CD40 ligation induced modest secretion of TNF-α and robust production of IL-12. Given that IL-12 has been reported to protect *in vitro*-derived DCs from apoptosis induced by co-culture with prostate cancer cells [23], we examined whether IL-12 could protect blood DCs from apoptosis induced by breast tumor-derived supernatants. Exogenous IL-12 significantly (*p *< 0.05) reduced blood DC apoptosis (Fig. 4b), indicating that IL-12 also has a protective role against tumor-induced apoptosis.

### Sustained expression of Bcl-2 correlates with protection

**Figure 5:**
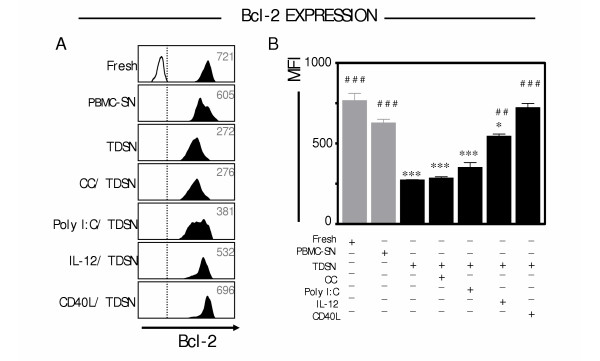
Effect of tumor-derived supernatant (TDSN) and stimulation on expression of Bcl-2. **(a)**Expression of Bcl-2 in blood dendritic cells (DCs) was determined: *ex vivo *(Fresh); following culture (24 h) in the presence of 50% (v/v) peripheral blood mononuclear cell-conditioned supernatant (PBMC-SN) or TDSN (MA11); or following stimulation (24 h) with a cytokine cocktail (CC), synthetic double-stranded RNA (poly I:C), IL-12 or CD40 ligand (CD40L) prior (24 h) to culture with 50% (v/v) TDSN. Representative histograms (filled) are shown with numbers indicating the mean fluorescence intensity (MFI) of Bcl-2 expression. The unfilled histogram shows staining with isotype control. **(b)**Summary of Bcl-2 expression data. Results show the average ± standard error of the mean of five independent experiments for which statistically significant differences compared to fresh cells (*, *p *< 0.05; ***, *p *< 0.001) or after incubation with TDSN (^##^, *p *< 0.01; ^###^, *p *< 0.001) are indicated.

Given that members of the Bcl-2 family of proteins are involved in the regulation of tumor-induced apoptosis in DCs derived i*n vitro *[7], we examined whether protection from TDSN-induced apoptosis could be mediated by modulation of the expression of the anti-apoptotic protein Bcl-2. We found that incubation of cells with TDSN, but not PBMC-SN, resulted in significant (*p *< 0.05) down-regulation of Bcl-2 expression in blood DCs (Fig. 5a,b). Interestingly, pre-incubation of cells with the CC or poly I:C did not significantly change the reduction of Bcl-2 expression caused by tumor supernatants (Fig. 5b). In contrast, pre-incubation with IL-12 or CD40L resulted in sustained expression of Bcl-2, abrogating the suppressive effect induced by the TDSN (Fig. 5b). In fact, comparable levels of Bcl-2 expression were observed on fresh samples or following culture with CD40L (Fig. 5b). These data suggest loss of Bcl-2 is involved in blood DC apoptosis induced by TDSN and that protection from TDSN-induced apoptosis (as shown for CD40L and IL-12) involves sustained expression of this molecule.

## Discussion

In this study, we document the presence of a significantly higher percentage of apoptotic blood DCs in patients with breast cancer compared to healthy volunteers, suggesting that in these patients, a higher proportion of blood DCs are programmed *in vivo *to undergo apoptosis. This phenomenon appeared to be related to their cancer as all patients were newly diagnosed (no prior therapy) and no comparable apoptosis was observed in blood DCs from healthy volunteers. Although previous reports have described increased apoptosis in tumor-infiltrating DCs in patients with melanoma and ovarian cancer [7], our findings clearly indicate that the inhibitory influence of the tumor extends far beyond the tumor microenvironment. In keeping with this, previous studies [16,17] have described spontaneous apoptosis of several mononuclear cell subsets (T and B lymphocytes, NK cells and monocytes) in blood of patients with different types of cancer, suggesting a rather generalized phenomenon. This, however, is the first description of spontaneous apoptosis in DCs from the peripheral blood of patients with cancer.

The physiological and clinical significance of blood DC apoptosis in patients with cancer is of substantial interest. Circulating DCs are essential for adequate immunity given that they continually replenish the pool of tissue-residing DCs and play a critical role in shaping immune responses *in vivo *[1]. Indeed, most circulating DCs appear to be *en route *from the bone marrow to peripheral and lymphoid tissues or from non-lymphoid tissues to the regional lymph nodes and spleen [24,25]. Given that apoptotic cells are rapidly cleared from the circulation by the reticulo-endothelial system, our observation of a higher fraction of blood DCs undergoing apoptosis in patients with breast cancer suggests increased turnover of these cells *in vivo*. If this assumption is correct, continual efforts to replace the pool of blood DCs from bone marrow would impose chronic stress on the immune system of breast cancer patients, resulting in a relative paucity of DCs in the circulation [15,26] as well as a failure to effectively replenish DCs that infiltrate breast tumor tissue [13,14] or in the ability of DC to migrate to lymphoid organs [12] for the initiation of T-cell immunity. Accordingly, in patients with operable breast carcinoma, blood DC numbers are significantly reduced over prolonged periods of time (approximately 48 weeks post surgery) independently of other blood cell counts (monocytes, neutrophils, platelets), suggesting diminished availability of DC precursors in these patients (A Pinzon-Charry *et al*, unpublished observations). Moreover, in a cohort of 35 patients with early (stage I and II) and advanced (stage IV) breast cancer (stage I, n = 17; stage II, n = 10; stage IV, n = 8), we found that reduction in blood DC numbers correlated with disease progression [27]. The resulting immune dysfunction would lead to reduced antitumor immunity [11] and, thus, tumor progression.

The increased pro-apoptotic effect of tumor supernatants demonstrated in this work may have a differential effect on the various DC subsets. In a separate study, we have carefully examined the DC compartment for various immune functions. We identified a population of Lin^-^HLADR^+ ^cells that is CD11c and CD123 negative that appears to be particularly resistant to apoptosis induced by supernatants derived from breast (MB231, MA11, MB435, SKBR3 and MCF7) as well as colon (LOVO) cancer cell lines. In contrast, Lin-HLA^-^DR^+^CD11c^+^CD123^+ ^DCs consistently undergo increased levels of apoptosis under the same conditions [28].

We therefore set out to directly confirm the role of tumor products in the induction of blood DC apoptosis in breast cancer. We found that supernatants derived from several breast cancer lines significantly reduced blood DC survival as assessed by Annexin-V, TUNEL and morphological analyses. Our findings on blood DCs confirm previous studies on *in vitro *generated monocyte-derived DCs wherein tumor products (IL-10, prostanoids, gangliosides or ceramides) induced marked levels of apoptosis [4,5,29]. Moreover, in view of the increased level of apoptosis in circulating DCs in patients with cancer described here, it is tempting to speculate that pro-apoptotic tumor products that regularly gain access to the peripheral circulation at high concentrations, such as IL-10 and gangliosides [30,31], could potentially impair viability of blood DCs *in vivo *[2].

From an immunotherapy perspective, our results are relevant in two ways. Firstly, because blood DCs have been proposed for use in cancer immunotherapy [9]. Blood DCs offer the theoretical advantage of being in their natural state of differentiation and presumably capable of stimulating immune responses in a more physiological manner. Apoptotic DCs, however, are ineffective at inducing immunity [32], which may explain, at least in part, the failure of blood DCs from breast cancer patients to capture antigens and generate adequate T-cell responses as described by us (Additional file 1) and others [11,12]. Secondly, because therapeutic DCs would be subject to suppression when re-introduced into patients and, thus, re-exposed to pro-apoptotic products derived from the tumor. In this regard, our results demonstrate that the addition of specific maturation stimuli can protect blood DCs from tumor-induced apoptosis, thus facilitating their survival and potential effectiveness.

Indeed, by comparing a range of stimuli available for clinical use, including inflammatory cytokines (CC), poly I:C and soluble CD40L, we found that the CC and poly I:C induced robust phenotypic maturation, but failed to protect blood DCs from apoptosis. These results imply that the upregulation of maturation and costimulatory molecules may have only minimal effect on DC survival. In contrast, CD40 stimulation induced strong phenotypic maturation, in addition to augmented IL-12 secretion and protected blood DCs from TDSN-induced apoptosis through sustained expression of the anti-apoptotic molecule Bcl-2. Similarly, exogenous IL-12 protected blood DCs from apoptosis through sustained expression of Bcl-2, suggesting that CD40L-induced protection could be mediated, at least in part, through IL-12 secretion.

Together with Toll-like receptor (TLR) interactions, DC survival induced by CD40L appears to be mediated by the activation of NF-κB transcription factor proteins [33]. In a mouse model, the beneficial effect of CD40 ligation has been related to the function of the anti-apoptotic protein Bcl-2, which counter-balances the apoptotic property of various DC maturation stimuli [34]. Our data support and advance this idea in that we identify IL-12 as playing a similar protective role. Further studies on the mechanisms involved in this process will provide better understanding of the homeostatic function of Bcl-2 in DC survival. Interestingly, TRANCE/RANKL, another modulator of NF-κB, has been implicated in prolonged DC survival [35], particularly after stimulation with CD40L [36]. A recent report correlated a high expression of RANKL in breast tumor cells with a decreased metastatic (bone) phenotype [37]. Considering our data, it is tempting to speculate that the joint effect of RANKL with CD40L leading to prolonged DC survival may prevent tumor growth; an additional argument for the potential benefit of CD40L linked with DC immunotherapy for breast cancer.

In models in which DCs are generated *in vitro*, CD40 stimulation can induce increased DC survival and IL-12 secretion [38], thus promoting IFN-γ production by T-helper cells [39] as well as tumor-specific cytotoxic responses [40]. These data strongly support the use of CD40L conditioning for DCs in cancer immunotherapy. Hitherto, one study has shown the potential of CD40L as an efficient stimulator of professional APC under clinically applicable conditions [41] and two ongoing cancer trials with CD40L-conditioned DCs [42] are awaiting completion. Our results demonstrate that *ex vivo *conditioning of blood DCs with CD40L can protect them from tumor-induced apoptosis and, thus, further support this approach.

## Conclusion

Our data demonstrate that in the peripheral circulation, more blood DCs are undergoing apoptosis in patients with breast cancer than in healthy donors. Given that no significant apoptosis was detected in samples from healthy donors, we propose that these cells are programmed *in vivo *to undergo apoptosis by a mechanism related to the presence of tumor products. Indeed, supernatants from breast cancer cell lines induce significant apoptosis of blood DCs *in vitro*. Moreover, we show that loss of expression of the anti-apoptotic molecule Bcl-2 is involved in apoptosis induced by breast cancer cell line supernatants and demonstrate that exogenous conditioning with CD40L (and IL-12) protects blood DCs from apoptosis through sustained expression of Bcl-2. Cumulatively, our findings support the use of exogenous conditioning of DCs to ensure their survival and, thus, may prove crucial in improving the efficacy of DC-based immunotherapies for cancer.

## Abbreviations

APC = allophycocyanin; CC = cytokine cocktail; DC = dendritic cell; FACS = fluorescence activated cell sorting; FITC = fluorescein isothiocyanate; FSC = Forward side characteristic; IL = interleukin; Lin = lineage markers; Lin^-^HLA-DR^+ ^= lineage negative HLA-DR positive; PBMC = peripheral blood mononuclear cell; PBMC-SN = PBMC-conditioned supernatant; PBS = phosphate-buffered saline; PE = phycoerythrin; PG = prostaglandin; SSC = side scatter characteristic; TDSN = tumor-derived supernatant; TNF = tumor necrosis factor; TLR = Toll-like receptor; ΔMFI = delta mean fluorescence intensity; 7-AAD = 7-Aminoactinomycin D.

## Competing interests

The authors declare that they have no competing interests.

## Authors' contributions

AP-C and JAL conceived and designed the study. AP-C and TM performed the laboratory studies. AP-C, CF and JAL enrolled the patients. AP-C, MAM, CS and JAL analyzed the data. All authors contributed to writing the paper.

## Supplementary Material

Additional file 1 A figure showing impaired uptake and allo-stimulatory capacity of blood DCs from patients with breast cancer. Click here for file
