# Supplementary material for: Spontaneous apoptosis of blood dendritic cells in patients with breast cancer
Source: Breast Cancer Res. 2005 Dec 16;8(1):R5. doi: 10.1186/bcr1361 (PMC1413992; doi:10.1186/bcr1361)
Supplement: Additional file 1 — A figure showing impaired uptake and allo-stimulatory capacity of blood DCs from patients with breast cancer. [file bcr1361-S1.pdf]

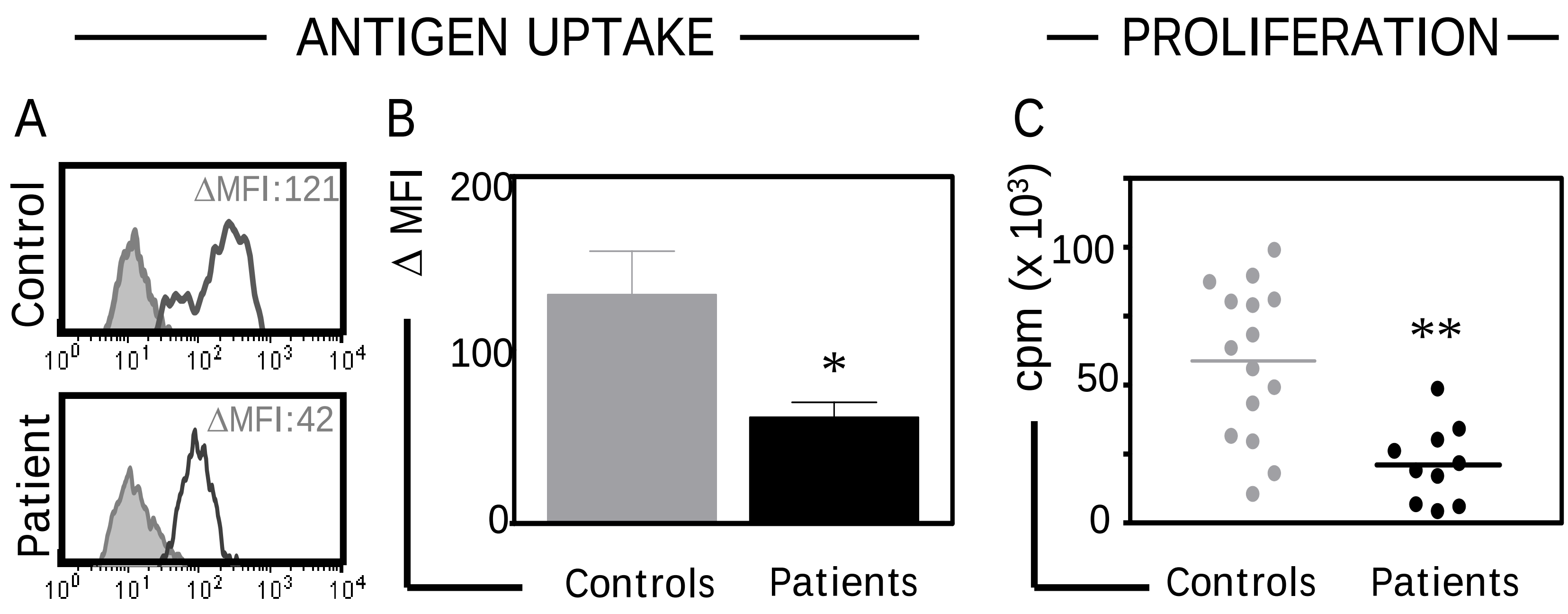

Supplementary Figure 1 Pinzon-Charry *et al*

Impaired uptake and allo-stimulatory capacity of blood DC from patients with breast cancer

(A) To estimate antigen uptake, PBMC isolated from patients with breast cancer (Stage I-II; *n=13*) or healthy volunteers (*n=15*) were incubated with soluble FITC-conjugated tetanus toxoid (0.5 mg/mL) at either 4°C or 37°C. Cells were extensively washed and uptake by blood DC (Lin^{-} HLA-DR^{+} cells) estimated by flow cytometry as the difference in mean fluorescence intensity (ΔMFI) between the test (37°C, empty histograms) and the negative control (4°C, filled histograms). In all experiments, each patient was tested in parallel with at least one healthy volunteer. Representative histograms are shown. **(B)** Summary of antigen uptake data. The average antigen uptake (ΔMFI ± standard error of the mean, SEM) by blood DC from all patients and healthy volunteers is shown accordingly. **(C)** To estimate allo-stimatory capacity, blood DC purified (99% purity; MoFlo Sorter) from breast cancer patients (Stage I-II; *n=10*) or age-matched healthy volunteers (*n=15*) were individually tested against allogeneic T-cells obtained from a panel of healthy volunteers (*n=3*). The pairs giving maximal responses are shown as means of triplicate measurements of ^{3}H-thymidine uptake at 1:30 DC:T ratio. Similar patterns of results were found for all DC:T ratios. Overall means are shown as horizontal lines and statistically significant differences between controls and patients are indicated * $p<0.05$, **$p<0.01$. Altogether, these data show significantly reduced antigen uptake and allo-stimulatory capacity of blood DC from breast cancer patients compared to blood DC from healthy volunteers.
